# Vesicular Trafficking and Signaling for Cytokine and Chemokine Secretion in Mast Cells

**DOI:** 10.3389/fimmu.2014.00453

**Published:** 2014-09-22

**Authors:** Ulrich Blank, Iris Karina Madera-Salcedo, Luca Danelli, Julien Claver, Neeraj Tiwari, Elizabeth Sánchez-Miranda, Genaro Vázquez-Victorio, Karla Alina Ramírez-Valadez, Marina Macias-Silva, Claudia González-Espinosa

**Affiliations:** ^1^INSERM UMRS 1149, Paris, France; ^2^CNRS ERL8252, Paris, France; ^3^Université Paris Diderot, Sorbonne Paris Cité, Laboratoire d’excellence INFLAMEX, Paris, France; ^4^Instituto de Fisiología Celular, Universidad Nacional Autónoma de México, México City, México; ^5^Departamento de Farmacobiología, Cinvestav, México City, México

**Keywords:** mast cells, signaling, vesicular trafficking, secretion, inflammation

## Abstract

Upon activation mast cells (MCs) secrete numerous inflammatory compounds stored in their cytoplasmic secretory granules by a process called anaphylactic degranulation, which is responsible for type I hypersensitivity responses. Prestored mediators include histamine and MC proteases but also some cytokines and growth factors making them available within minutes for a maximal biological effect. Degranulation is followed by the *de novo* synthesis of lipid mediators such as prostaglandins and leukotrienes as well as a vast array of cytokines, chemokines, and growth factors, which are responsible for late phase inflammatory responses. While lipid mediators diffuse freely out of the cell through lipid bilayers, both anaphylactic degranulation and secretion of cytokines, chemokines, and growth factors depends on highly regulated vesicular trafficking steps that occur along the secretory pathway starting with the translocation of proteins to the endoplasmic reticulum. Vesicular trafficking in MCs also intersects with endocytic routes, notably to form specialized cytoplasmic granules called secretory lysosomes. Some of the mediators like histamine reach granules via specific vesicular monoamine transporters directly from the cytoplasm. In this review, we try to summarize the available data on granule biogenesis and signaling events that coordinate the complex steps that lead to the release of the inflammatory mediators from the various vesicular carriers in MCs.

## Introduction

Mast Cells (MCs) are tissue cells that are well known effectors in IgE-mediated allergic or anti-parasitic responses, but research in the last two decades has revealed that they are also important participants in innate immunity and inflammation (1, 2). One of their prime functions is to release a large array of inflammatory mediators, which mediate tissue responses, participate in immunoregulatory and inflammatory processes as well as tissue remodeling (2–5). These mediators include compounds prestored in a release-ready form in their cytoplasmic secretory granules (SGs) such as histamine and MC specific proteases. MC also secrete newly synthesized lipid mediators such as leukotrienes or prostaglandines or a variety of cytokines, chemokines, and growth factors (3, 6). While synthesized lipid compounds freely diffuse across the membrane, the release of protein products depends on vesicular carriers and membrane fusion (7, 8).

Study of the biologic function and involved signaling pathways engaged by various membrane receptors has evidenced that MC can release either their full array of mediators or just some of them under particular conditions of stimulation (9). For example, while stimulation through the IgE Fc receptor (FcεRI) generally leads to a full response, stimulation through Toll-like receptor 2 and 4 (TLR2 and TLR4) or through some cytokine receptors induces the selective release of newly synthesized chemokines and cytokines in the absence of degranulation (9). The release characteristics also depend on the strength of stimulus as, for example, after weak stimulation (low antigen concentration in case of FcεRI) the release of chemokines over cytokines is favored (10). This is due to the engagement of different signaling pathways that at least in part imply engagement of distinct Src-related kinases (11).

Research in recent years on secretory mechanisms after stimulation has mostly focused on the degranulation process that concerns the release of mediators stored in cytoplasmic granules, which amongst many other compounds includes also certain cytokines (4). At present, little is known on the vesicular trafficking of newly synthesized cytokines and chemokines and its positive and/or negative regulation. Yet, it is clear that like secretion from cytoplasmic granules the release of newly synthesized chemokines, cytokines, and growth factors must be tightly regulated to ensure an appropriate biological response. Here, we review some of the important aspects of the vesicular trafficking mechanisms and their involvement in MC cytokine and chemokine secretion.

## Secretory Pathways in MC

**Figure 1:**
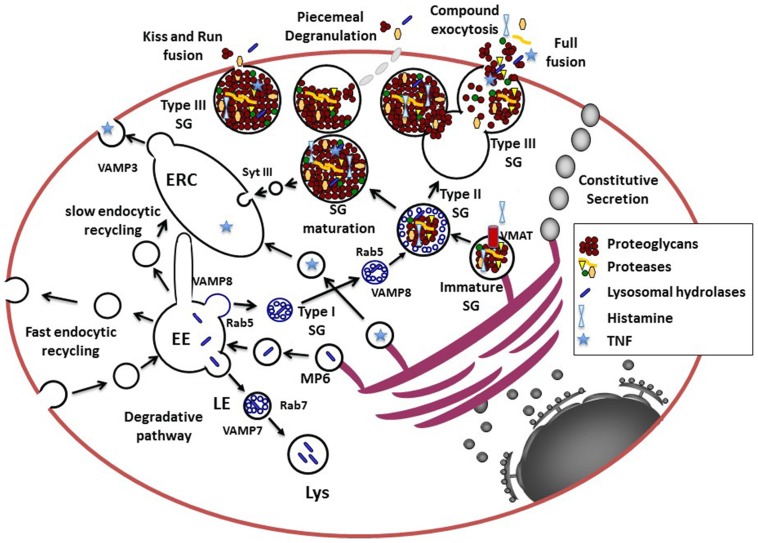
**Model for the secretory pathways in MC**. Newly synthesized proteins destined for secretion enter the secretory pathway at the rough ER. After passage through the Golgi where posttranslational modifications occur, they reach the trans-Golgi network (TGN), which functions as a sorting hub depending on various structural motifs. For example, GPI-anchored or *N*-and *O*-glycosylated proteins enter the constitutive secretory pathway for trafficking to the cell surface. Proteins destined for the endosomal–lysosomal pathway are modified by Mannose-6 phosphate (MP6) for recognition by the Mannose-6-phosphate receptor that via an acidic cluster/dileucine motif is delivered to the early endosome (EE). Together with endocytosed proteins destined for degradation they are further sorted to late endosomes (LE) forming multivesicular structures and then fuse with lysosomes (Lys) via a VAMP-7 and Rab7-dependent pathway. Some proteins, like for example, newly synthesized TNF may be sorted into vesicles to reach the endocytic recycling compartment (ERC) from where it could be secreted. The ERC may serve as an additional sorting hub for the exocytosis and recycling of proteins via retrograde pathways (not shown in this figure). The cytoplasmic SG, which contain proteins destined for the regulated secretory pathway for release upon stimulation in MC and other hematopoietic cells are so-called mixed type organelles or secretory lysosomes carrying features of endosomes and lysosomes. Proteins destined for SG may leave the TGN at sites where larger protein aggregates are formed possibly via association to highly negatively charged proteoglycans. The immature SG formed may then rapidly fuse with carriers (type I SG) containing small intraluminal vesicles derived from the early endosome via a Rab5 and VAMP-8 dependent mechanism to form type II SG. Some inflammatory mediators like histamine incorporates into SG via specific vesicular monoamine transporters (VMAT). SG then undergo a maturation process, which generates mature type III SG by the retrieving cargo of missorted proteins and intraluminal vesicles to the ERC. Pre-formed inflammatory mediators can then be released via several types of fusion processes including transient kiss-and-run fusion, piecemeal degranulation, or multigranular/compound exocytosis, which involves full fusion and collapse of SG. It is possible that during stimulation all types of granules (type I, II, and III) are fusion competent as suggested by the fact that cultured cells with less mature granules can be stimulated for release.

In MC, like in any other cell type, the secretory pathway (Figure 1) starts with the translocation of nascent signal-peptide containing proteins at the rough endoplasmic reticulum (ER) after recognition by the cytosolic signal-recognition particle (SRP) ribonucleoprotein complex, which binds to the ER-localized SRP-receptor (12). From the ER, the proteins sequentially travel through the Golgi stacks via a still not completely understood process involving either Golgi cisternal maturation or vesicular transport carriers (13). While passing the Golgi stacks posttranslational modifications occurs, such as for example, glycosylation, and proteins finally reach the Trans-Golgi network (TGN). The TGN functions as a sorting hub for protein trafficking (14–16). By default, via the constitutive secretory pathway, glycosylated or glycosylphosphatidylinositol (GPI)-anchored proteins are delivered to the plasma-membrane (PM) via tubular–vesicular carriers that bud off from the ER by a well regulated process involving motor proteins and fission processes and then fuse with the PM (16). However, in the presence of specific sorting signals (sorting by exit) proteins may be routed to other intracellular compartments. For example, within the Golgi apparatus, lysosomal hydrolases acquire a mannose-6-phosphate (M6P) moiety (17). M6P is then recognized by the M6P receptor (M6PR) at the TGN. An acidic cluster/dileucine motif in the cytoplasmic tail of the M6PRs then serves as a sorting signal for specific adaptor proteins (such as AP-1) allowing packing into clathrin-coated transport vesicles targeting them to early endosomes (16). Within early endosomes, the acidic environment of the endosomal lumen releases hydrolases from the M6PR. After maturation into late endosomes a process that is accompanied by the generation of intraluminal vesicles via the endosomal sorting complexes required for transport (ESCRT) machinery (18) the late endosomes fuse with existing lysosomes, where hydrolases and other proteins like proteases accumulate and get activated to degrade proteins.

Another important pathway is the regulated secretory pathway. Specialized secretory cells such as endocrine, neuronal, exocrine, but also hematopoietic cells have the capacity of regulated secretion liberating secretory cargo stored in cytoplasmic granules following arrival of a stimulus (14). While endocrine, neuronal, and exocrine cells are known to form specialized SGs, cells from the hematopoietic lineage, but also some other cells such as melanocytes, possess mixed type organelles often called secretory lysosomes due to their close connection between the endocytic and exocytic pathway (19). They are characterized by the presence of lysosomal enzymes as well as markers of lysosomal origin such as CD63/LAMP-3, and LIMP IV/5G10 antigen (17). They often are also altered in patients with lysosomal storage diseases carrying mutations in a variety of genes involved in lysosome biogenesis (19).

The biogenesis of SG has been well worked out in neuroendocrine cells (15). In these cells, proteins targeted to the regulated secretory pathway become associated with specialized regions of the TGN forming larger aggregates that eventually associate with other soluble or membrane-localized cargo protein (sorting by entry) (14). After budding immature SG are formed that, via a series of homotypic fusion events, give rise to mature SG. This process is coupled to the removal of non-secretory missorted cargo via clathrin-coated vesicles (sorting by retention) and condensation, the latter being at least partially favored by the acidic environment in the granules maintained actively by proton pumps (5). In contrast to the well-characterized granule biogenesis in neuroendocrine cells, the biogenesis of secretory lysosomes is less clear. While melanosomes, for example, seem to derive from early or sorting endosomes with few small intraluminal vesicles (20), granules from lytic T cells are likely modified lysosomes containing a ring of multivesicular bodies, which derive from the fusion of late endosomes, surrounding a dense core that further matures upon T cell activation (19–21). The dense cores might derive, like for conventional SG, from the TGN. It is possible that proteoglycans may play an important role by their capacity to selectively aggregate and attract regulatory secretory proteins via charged interaction thereby serving as a nucleation point for sorting (22). In favor, experiments carried out with proteoglycan deficient hematopoietic cells, including MC, all show serious defects in granule biogenesis, protease content, and maturation (5). Additional studies showed that SG containing at least some of the granule proteases and mediators could still be formed even in the absence of proteoglycans leaving open the possibility of proteoglycan-independent formation of immature SG (23). However, the fact that all these SGs are morphologically altered lacking an electron dense core and crucial enzymes favor an important role of proteoglycans in physiological SG formation.

In MC, electron microscopy studies (24) have allowed to define three types of SGs: (1) type I SG likely representing matured endosomal/lysosomal organelles with numerous intraluminal vesicles that are rapidly accessible to endocytic tracers, (2) type II SG, which like in cytotoxic T cells contain a dense protein core surrounded by multivesicular bodies; these granules are also rapidly accessible to endocytic tracers, and (3) type III SG containing essentially electron dense material no more accessible to endocytic tracers. Likely, type III SGs are generated by a maturation process that may involve Synaptotagmin III (SytIII) regulating recycling and delivery of cargo to the endocytic recycling compartment (ERC) during granule maturation (25). This suggests that the ERC could be an important intermediate in granule recycling and maturation. The nature of the involved endocytic compartment is also not completely clear, but recent data have indicated an important role of the early endosomal marker Rab5 and VAMP-8 in granule size determination (26). This suggests that multivesicular bodies or type I SG may not derive from late endosomes but could directly form at the early endosome and fuse with Golgi-derived specialized SGs in homotypic fusion events (26). A possible role of SytIX in this process has been proposed as it excludes proteins destined for recycling to reach the SG (27). However, the fact that in human MC VAMP-7 serves as a soluble *N*-ethylmaleimide-sensitive factor attachment protein receptor (SNARE) for release of SG may suggest that genuine late endosomes may also take part in the generation of SG (28). Granule maturation is a long process that can take several months in MC, as indicated by data (29) showing that MC SG get gradually enriched with incoming cargo such as proteoglycans, MC proteases, but also histamine and serotonin, the latter of which being transported via vesicular monoamine transporter (VMAT) systems directly from the cytoplasm (5). Interestingly, these positively charged molecules also contribute to granule homeostasis as in their absence SG maturation is affected as shown for histamine and MC proteases (5, 30). In addition to these classical sorting pathways, the ERC, initially characterized for its role in the slow pathway of Transferrin recycling, has also gained attention as a new sorting hub actively sorting cytokines for release as demonstrated in macrophages (31).

## Anaphylactic Degranulation

### Prestored inflammatory mediators

MC are the effector cells of type I hypersensitivity reactions, which involves the release within a few minutes of cytoplasmic granular content into the surroundings after stimulation, a process often called anaphylactic degranulation (32). A potent stimulus for degranulation is the crosslinking of IgE bound to FcεRI by multivalent antigens or allergens (3). Indeed, one distinctive features of MC is that they contain numerous SGs in their cytoplasm (32, 33). These granules are filled with different inflammatory compounds, many of which are bound to a highly charged anionic gel matrix composed of proteoglycans such as heparin or chondroitin sulfate, which differ among the different MC types and species (34). The presence of these proteoglycans attributes the specific staining properties of MC with cationic dyes such as toluidine blue enabling the classical metachromatic staining of MC in tissues, which have led to the discovery of these cells by Paul Ehrlich in the nineteenth century (35). Well-known mediators bound to these proteoglycans are cationic amines such as histamine or serotonin, but also proteases such as tryptase, chymase, and carboxypeptidase [for a complete review see for example (4, 36)]. The formation of such a gel matrix allows their tight packaging and is thermodynamically advantageous avoiding osmotic work (37).

When MCs are stimulated, up to 100% of granular content can be released in a single stimulation event within minutes enabling a maximal biological effect in its immediate vicinity, but also often with systemic effects as for example, occurring during a generalized anaphylactic shock (38, 39). This degranulation is made possible by a special type of exocytosis mechanism often called compound exocytosis involving the fusion of PM proximal granules with the PM followed by the sequential fusion with granules lying deeper in the cytoplasm (Figure 1). Although such sequential fusion events have been shown by electron microscopy studies (33), a multigranular fusion mode that leads to the fusion of large intracellular fused granules with the PM has often also been observed (40). Degranulation involves the extrusion of the whole condensed gel matrix, which is accompanied by a drastic swelling due to their hydration (41). The change in pH after release from the acidic milieu in the granule to the neutral pH in tissues allows the dissociation of cationic inflammatory mediators such as histamine and MC proteases (5). In addition to this anaphylactic degranulation mechanism, granular content can also be released in small portions by a mechanism called piecemeal degranulation (PMD), characterized by the gradual emptying of MC SG over much longer periods of time, but also kiss-and-run fusion involving only a short opening and contact with the external environment (see below).

**Figure 2:**
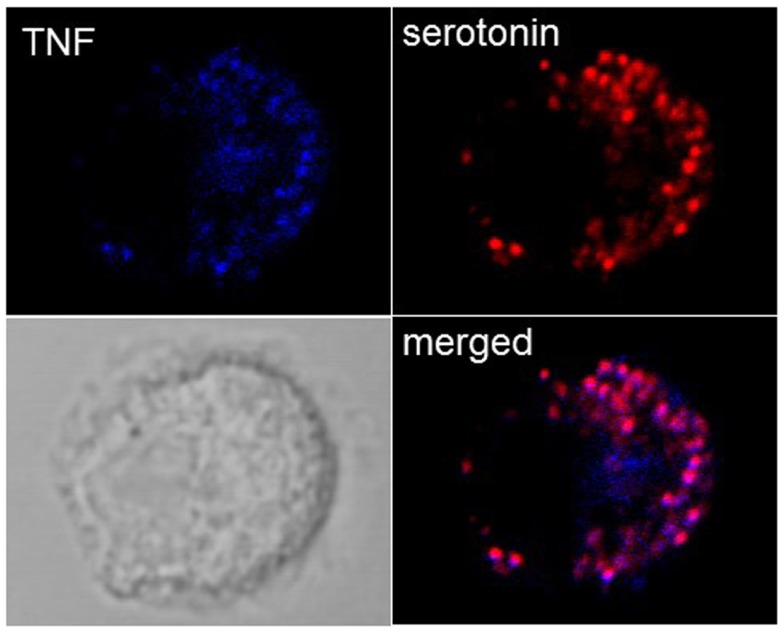
**Localization of TNF in SG in cultured BMMC**. Colocalization of TNF (blue) with the SG markers serotonin (red) were analyzed in non-stimulated BMMC using confocal microscopy. Representative single optical sections and overlay (Merge) and the DIC images are shown. Note that in a single field only few cells show such TNF SG granular staining.

While it was thought initially that cytokines are released only after new synthesis, in groundbreaking discoveries, Galli and coworkers (42) found that MC in addition to releasing newly synthesized TNF were able to store TNF in SG from where it could be released through anaphylactic degranulation. In particular, highly differentiated tissue MC such as peritoneal MC in mice were found to contain substantial amounts of TNF in their SG, while this was less the case in cultured cells. In fact, in some cultured MC lines, such as for example PT18 or RBL-2H3 (42, 43), no prestored TNF is detectable while this amount seems variable for other lines or primary cultured MC such as BMMC. Our own experiments in BMMC show that they contain detectable TNF that colocalizes with SG markers (Figure 2), but this concerns only a small fraction of cells, while other cells clearly express only classical granule markers such as serotonin or proteases. It is therefore possible that TNF can accumulate only in mature granules. As a possible mechanism a specific sorting signal based on N-glycosylation transporting TNF to the endosomal system was proposed (44). In human MC, where TNF is not glycosylated, TNF became enriched in SG after transient exposure on the PM and re-endocytosis (45). Besides TNF, a series of other cytokines including for example, IL-4, IL-5, IL-13, and vascular endothelial growth factor (VEGF) have been reported to be prestored in MC SG (4). Prestored cytokines have also be found in other cells of hematopoietic origin including eosinophils, platelets, and neutrophils (46–49).

### Early signaling pathways in anaphylactic degranulation

Anaphylactic degranulation can be induced via immunoreceptors, the FcεRI as a prototype (3), but MC can also be stimulated via IgG receptors under certain conditions (50). Furthermore, MCs express a whole variety of other receptors that can promote anaphylactic degranulation in particular certain G protein coupled receptors (GPCRs) such as receptors for complement peptides C5a and C3a, receptors for neuropeptides or receptor for certain inflammatory peptides such as for example endothelin (4). Independent of the receptor type, they all can induce a calcium signal and the activation of protein kinase C (PKC), in particular PKCα/β, which are necessary for anaphylactic degranulation to occur (3, 51). Signals which do not induce a substantial calcium response, such as for examples those generated via LPS and Toll-like receptors, although inducing the secretion of newly synthesized cytokines or chemokines do not induce anaphylactic degranulation.

Some recent reviews describe in detail the main molecular events following FcεRI crosslinking in MC (6, 51). Briefly, after antigen-dependent activation of FcεRI receptor, Src family kinases Lyn and Fyn are activated causing phosphorylation of FcεRIβ and FcεRIγ chains ITAMs (Figure 4). Receptor activation opens membrane calcium channels causing a receptor-operated calcium entry (ROCE). Lyn kinase activation leads to the recruitment and activation of Syk kinase. Syk, in turn, phosphorylates the adapters LAT, SLP76, Gab2, and NTAL, among others. Those adapters participate in the assembly of large signaling complexes that include molecules such as Bruton’s tyrosine kinase (BTK), which activates phospholipase Cγ (PLCγ) cooperatively with Syk. PLCγ activation induces diacylglycerol (DAG) production and inositol-1,4,5-trisphosphate (InsP3)-induced Ca^2+^ mobilization. Emptiness of intracellular calcium stores leads to the activation of a store-activated calcium entry (SOCE) to replenish internal stores. DAG and Ca^2+^ induce the activation of classical isoforms of PKC, PKCα, and β. On a complementary pathway, Fyn kinase phosphorylates Gab2 leading to the activation of phosphoinositide 3 kinase (PI3K). This results in activation of the PI3K-dependent protein kinase I (PDK1) and PKCδ. PI3K activation induces Phospholipase D (PLD) and Akt activation, the latter favoring MC survival and mTOR activation (51, 52). The Lyn pathway is essential for Ca^2+^ signaling, whereas the Fyn-dependent pathway is required for degranulation and maintenance of Ca^2+^ mobilization (53).

### Late signaling events in anaphylactic degranulation

**Figure 3:**
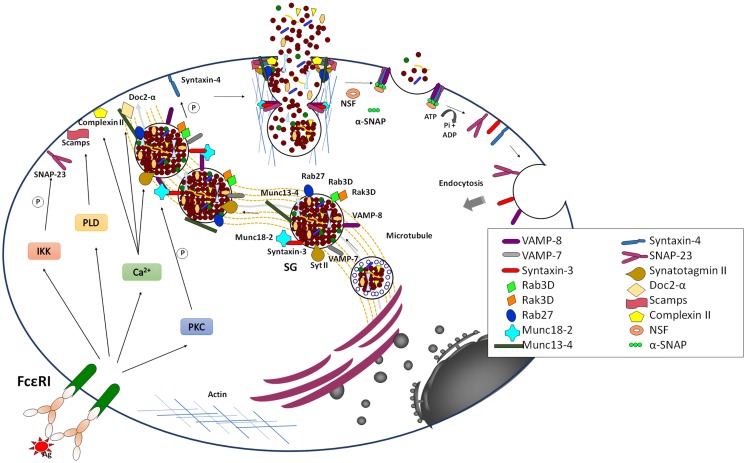
**Late signaling events in the anaphylactic degranulation**. Aggregation of IgE bound to FcεRI leads to the generation of elevated levels of intracellular Ca^2+^ and the activation of PKC. In parallel, it also leads to the activation of PLD and IκB Kinase (IKK). Together they trigger anaphylactic degranulation, which involves SG–SG and SG–PM fusion events. Fusion is mediated by SNARE proteins that lie on opposing membrane. The formation of a stable well-characterized trans-SNARE complex in MC comprises the v-SNARES VAMP-8 that pairs with the two t-SNAREs, Syntaxin 4, and SNAP-23. During SG–SG fusion SNAP-23 relocates to the cytoplasm, which may be characteristic for the compound/multigranular fusion mode in MC. The SG-localized t-SNARE Syntaxin 3 may be specifically implicated in SG–SG fusion events and in a manner opposite to SNAP-23 translocates to the PM upon activation. Other v-SNAREs like VAMP-2 and VAMP-7 might also play a role in anaphylactic degranulation, which may indicate redundance or heterogeneity of the MC secretory compartment. The formation of SNARE complexes is modulated by multiple accessories proteins such as Munc18-2, Synaptotagmin II, Complexin, SCAMP, Doc2α, Rab27A and B, and Munc13-4 that allow to connect the fusion machinery to early signaling events to relieve existing fusion clamps and to coordinate the docking, thethering, and fusion events as well as the connection to cytoskeletal reorganization and transport mechanisms. Fusion is closely coupled to endocytosis of membrane lipids and proteins. The individual SNARE proteins are reconstituted by an energy-requiring step, which is mediated by the NSF ATPase via the α-SNAP adaptor protein.

In addition to calcium and PKC, many different actors that are involved in the control of fusion during degranulation have been recently described (Figure 3). These include the highly conserved SNARE membrane fusion proteins (54–57). SNAREs can be divided into vesicular (v-SNARE) and target (t-SNAREs) localized, respectively, on opposing donor and acceptor membranes. They contain in their primary structure and about 60 aa α-helical SNARE motif, which upon arrival of the appropriate stimulus, can zipper to form a tight tetrameric trans-SNARE complex (composed of one v-SNARE and either two or three t-SNAREs, depending on the number of contained SNARE motifs) that drives the merger of lipid bilayers. After fusion SNAREs are disassembled under energy consumption by the ATPase *N*-ethylmaleimide-sensitive factor (NSF), which becomes recruited to the SNARE complex via an adaptor called Soluble NSF Attachment Protein (SNAP).

While, SNARE complex formation and fusion can be reconstituted into liposomes in the absence of any additional protein (58), the process is extremely slow indicating additional control mechanisms. In living cells, numerous accessory proteins have been found to regulate membrane fusion including Sec1/Munc18 (SM)-, Munc13-, Rab-, complexin-, and Synaptotagmin (Syt)-family members (56, 59). Together, they are responsible for the priming, tethering, and docking to ensure specificity and efficiency of the fusion process. They also allow to connect the fusion machinery to cell stimulation relieving existing fusion clamps. Many were initially characterized in neuronal secretion at the synapse. Although, in general, the basic principles described may apply to secretion in other cells, differences clearly exist. In particular, vesicular release at the synapse is set up for an extremely fast (within milliseconds) release followed by a fast recycling process at specific active zones requiring likely additional proteins such as voltage-gated calcium channels or proteins involved in vesicle docking such as RIM (59). In other cells, such as MC the process is generally more dynamic and might require a more extensive crosstalk with the cytoskeleton for transport mechanisms that does not occur at specific active zones. Furthermore, as mentioned above, they involve also granule–granule fusion events to obtain a maximal release within the scale of a few minutes. Some of the differences are summarized in Table 1.

**Table 1:** **Comparison of exocytosis in mast cells and neurons**.

Parameter	Mast cells	Neurons
Granules	Secretory lysosomes	Specialized secretory granules
Granule size	300–1000 nm	50 nm
Granule number	Up to 1000/cell	200–500/nerve terminal
Ca^2+^ requirements	1 μM	200 μM
PKC requirement	Yes	No
Time frame of exocytosis	Minutes	Milliseconds
Recycling time	Long (hours to days)	Very short (seconds to minutes)
Site of release	Multidirectional^a^	Active zone
Release characteristics	One single release, up to 100% of total granular content; multigranular/compound mode	Multiple release one granule per fusion event

*^a^Note that under specific local conditions of stimulation, mast cells are able to deliver SG content within specific zones*.

Several of the early studies in MC provided convincing evidence that, like in neurons, fusion implies SNARE proteins (60–63). Work from several laboratories has allowed to define a central SNARE complex composed of the PM-localized t-SNAREs SNAP-23 and Syntaxin 4 as well as the vesicular localized VAMP-8 (64–66). Interestingly, SNAP-23 was found to relocate to the cytoplasm in degranulation channels following activation, which could be a specific characteristic of the compound/multigranular mode of fusion as it enables granule lying deeper inside to get access to this PM SNARE (60). Furthermore, the implication of VAMP-8, which was initially described as an endosomal SNARE called endobrevin (67) was a clear indication for the close connection of the granular compartment with the endosomal/lysosomal system. In addition to these three SNAREs other possible SNAREs implicated include Syntaxin 3, VAMP-7, and VAMP-2 (28, 66, 68–70). In murine, but not in human MC (71), Syntaxin 3 is localized to SG and translocates to the PM, which might in a manner analogous to SNAP-23 be a characteristics of the compound/multigranular mode of fusion (70). However, the association with other SNARE proteins like Vti1b involved in vesicular fusion events is also possible (55). The fact that several types of SNARE complexes are implicated in membrane fusion in MC could be in agreement with the heterogeneity of the secretory compartment.

In addition to cognate SNARE proteins anaphylactic degranulation is regulated by an array of additional factors and signaling events. They include small GTPases known to regulate and coordinate discrete steps along the vesicular trafficking (72). A functional screen comprising 44 different Rab GTPases showed that many Rabs can affect either antigen and/or ionomycin/PMA triggered anaphylactic degranulation (73). The best characterized Rabs are the Rab27A and B isoforms. These studies indicate that while Rab27A may play a role in regulating the cortical actin disassembly limiting access of SG to the membrane, the role of Rab27B or the combined action of Rab27A and B facilitates degranulation by switching granules from microtubule-dependent movement to F-actin-dependent docking (74, 75). Indeed, degranulation is also accompanied by important remodeling of the actin cytoskeleton, which may favor the access to the PM (3). The docking step may imply another important factor, Munc13-4, that interacts with Rab27. Indeed, it was shown in MC that the interaction between both partners was required to correctly tether SGs at the PM and to prime them for fusion (75–77). Controversial data have been obtained with regard to the role of Rab3 isoforms. While initial data showed a potential role of Rab3A (78), we found that this isoform was not targeted to membranes in RBL MC and that its overexpression had no effect (79, 80). Rab3D, on the contrary, was SG-localized (81). Overexpression of wild-type and a constitutively active Rab3D mutant affected exocytosis (79). However, this was not confirmed in MC obtained from Rab3D knock-out animals although compensatory mechanisms by other Rab3 isoforms have not been examined (82).

Sec1/Munc18 protein family members are another type of fusion accessory proteins. They include three Munc18 isoforms implicated in exocytosis and some other family members involved in intracellular trafficking steps (56). Initial analysis of the neuronal isoform Munc18-1 suggested that it might be a negative regulator of fusion by its capacity to bind to particular syntaxin SNAREs preventing the binding of cognate SNARE partners. However, analysis of knock-out mice showed that its absence completely blunted synaptic transmission advocating a positive regulatory function (56). Later on this was explained functionally by data showing that during exocytosis Munc18-1 switches its binding mode to bind the assembled SNARE complex thereby aiding the fusion process probably by electrostatic interactions with the membrane for fusion pore expansion (56, 83, 84). MC do not express protein of the neuronal isoform Munc18-1 but express ubiquitious Munc18-2 and Munc18-3 (85, 86) and Munc18-1 does not seem to play a role (87). No functional data have been obtained for Munc18-3, but several studies by different authors have clearly shown that the Syntaxin 3 binding protein Munc18-2 acts as a positive regulator of fusion as in its absence exocytosis is compromised in MC (70, 88, 89) but also in other hematopoietic cells (90). Further functional studies indicated that knock-down of both Syntaxin 3 and Munc18-2 yielded additive inhibitory effects on exocytosis suggesting that Munc18-2 besides fusion might regulate additional steps (70). Video imaging of SG movement indicated that Munc18-2 affected SG translocation to the membrane, likely through its dynamic interactions with the microtubule cytoskeleton (70). Munc18-2 may also represent a possible target for the action of protein PKC (8, 91). As data in chromaffin cells already suggested a docking effect (92), it may be possible that SG docking may not be static, but rather a dynamic process that needs to be maintained via cytoskeletal interactions. In agreement, the neuronal Munc18-1 isoform has also be found to bind to the Kinesin-1 adaptor protein, fasciculation, and elongation protein zeta-1 (FEZ1) (93).

Secretory carrier membrane proteins (SCAMPs), a family of ubiquitous membrane proteins of transport vesicles (94) has also been shown to regulate fusion. These tetraspanins contain a short conserved segment (E-peptide) between the second and third transmembrane domain. MCs express three SCAMP isoforms, with SCAMP1 and SCAMP2 being most highly expressed. Introduction of the E-peptide interfered with degranulation in permeabilized MC (95, 96). SCAMPs may act at the final fusion step in agreement with data showing that genetic deficiency of SCAMP2 causes an apparent defect in forming stable fusion pores that may depend on Arf6-stimulated PLD activity (94). PLD was indeed proposed as an effector in MC exocytosis (97).

Several effectors molecules have been described that connect the fusion apparatus to Ca^2+^ signaling. This includes Syt family proteins, which are single transmembrane proteins with tandem calcium-binding C2 domains (termed C2A and C2B). Calcium-binding promotes oligomerization and membrane phospholipid-binding as well as the interaction with the SNARE complex, allowing the formation of a quaternary SNARE-Syt-Ca^2+^-phospholipid (SSCAP) complex driving of lipid bilayer mixing (98). While, neuronal expressed SytI was initially characterized as a possible sensor (99), recent studies in knock-out mice provided a clear evidence for the role of SG-localized SytII as the relevant Ca^2+^ sensor in MC (100). MC deficient in SytII showed a severe defect in both lysosomal β-hexosaminidase and histamine release and conferred to these mice a strongly decreased passive cutaneous anaphylaxis reaction (100). Although in addition to SytII, other Syt isoforms are expressed in MC, they do not seem to be directly involved in the fusion process but rather may regulate other intracellular trafficking steps (7). SytII may act in concert with ComplexinII (101, 102), a protein that binds to assembled SNARE complexes and that has been described in neurons to function as a fusion clamp. However, data from knock-out mice also support a positive action on exocytosis involving sequences adjacent to the SNARE-complex-binding domain. It seems that its interaction with the SNARE complex generates a metastable state that serves as a substrate for Syt following Ca^2+^-influx with the induced rearrangement and cooperative interactions promoting fusion (56, 59).

In addition to Syt, Doc2 adaptors also contain tandem calcium-binding C2A and C2B domains the former of which exhibiting calcium-dependent phospholipid-binding activity thought to be important for regulated exocytosis through possible interactions with Munc13 isoforms as well as partly assembled SNARE complexes (103). MCs express the Doc2α isoform thought to be neuronal-specific, while the ubiquitous Doc2β isoform was not expressed (104). Doc2α-deficient MC showed a marked defect in degranulation and formed a tripartite complex with Munc13-4 and Rab27 suggesting that it could play a role in vesicle priming (104). Another possible Ca^2+^-dependent process involves a kinase activity called Rak3D (from Rab3D-associated kinase) that was associated with Rab3D (105). Rak3D phosphorylated Syntaxin 4, but not Syntaxin 2 and 3 in its N-terminus, which prevented binding of the SNARE partner SNAP-23 suggesting that it could act as a fusion block. In agreement, the association with Rab3D decreased upon stimulation in a calcium-dependent manner. While phosphorylation of Syntaxin 4 was fusion inhibiting, phosphorylation of the other t-SNARE protein SNAP-23 rather seemed to be fusion promoting. This involved IκB kinase (IKK) also known to regulate the phosphorylation of IκB, which induces nuclear translocation of the NF-κB transcription factor (106). Indeed, in activated MC IKK phosphorylated a small fraction (~10%) of SNAP-23 on Ser95/Ser120 within its cysteine-rich linker region. In the absence of IKK, degranulation and anaphylactic responses were impaired. This was associated with decreased SNARE complex formation indicating a role for SNAP-23 phosphorylation in fusion.

## Other Forms of Degranulation

### Piecemeal degranulation

Piecemeal degranulation is a mode of exocytosis characterized by loss of granule content in the absence of clearly observable granule–granule or granule–PM fusion events. PM is associated with the generation of small vesicles ranging from 30 to 150 nm budding from large SGs (107, 108) (Figure 1). Empty spaces of lost secretory particles are recognizable inside the SG, without evidence for changes in the size of the granule membrane. The mechanism behind PMD is known as the “shuttling vesicle” hypothesis (109). According to this, the transport of granule constituents out the cell is mediated by vesicles shuttling from the granule compartment to the PM. Electronic microscopy has shown, in fact, that vesicular transport granule content is associated with this type of secretion (110). Thus, an outwarding flow of cytoplasmic vesicles loaded with granule materials effects granule depletion during PMD. Vesicles containing part of the granule content buds from the granule membrane and move through the cytoplasm and fuse with the PM, leading to content discharge. In a closely coupled inward flow, endocytotic vesicles are retrieved from the PM and traverse the cytoplasm to fuse with granules. If the rate and amount of vesicular traffic are balanced, granules and PM will maintain constant size. The most important characteristic of PMD is the fact that it seems to be modulated in different steps and by different stimulants. Strongly enhanced PMD was observed in mice overexpressing IL-4 resulting in eyelid lesions and enhanced fibrosis (111) or in human MC after stimulation with IL-1 (112). PMD has been studied in a number of different immune cells (47) but also enteroendocrine cells of the gastrointestinal tract, chromaffin cells of the adrenal medulla and chief cells of the parathyroid gland (108).

Alternatively to the shuttling vesicle hypothesis, PMD could also be the results of kiss-and-run fusion, which also result in partial vesicle emptying (see below).

### Kiss-and-run exocytosis

Kiss-and-run exocytosis refers to a special type of secretion in which a fusion pore opens and closes during vesicle fusion with the PM, allowing the release of some amount of granule content without full vesicle collapse (113). Kiss-and-run exocytosis (also known as “cavicapture”) has been reported in PC12 cells (114), chromaffin cells (115), and insulin secreting pancreatic beta-cells (MIN6)-cells (116). In MC, initial classical patch clamp studies led to the observation of capacitance changes in the PM due to exocytosis of individual granule contents after GTP addition and, interestingly, this event seemed to be independent of Ca^2+^ mobilization (117). Later, a series of experiments using peritoneal MC from beige mice demonstrated “capacitance flickering” after intracellular administration of GTPγS, suggesting rapid and reversible steps in membrane fusion due to the opening and closing of a fusion pore, narrow enough to retard the escape of some molecules (118) but leading to the leakage of some soluble granule components. Besides these electrophysiological measurements kiss-and-run fusion in MC could also be observed directly in multicolor multiphoton fluorescence microscopy in histamine loaded cells through the pH cycling that occurs after a release event. In these studies using a tumor MC line the authors found that kiss-and-run fusion occurs actually very frequently accounting for about one-third of all fusion events in MC (119).

The choice between kiss-and-run and full fusion of vesicles may depend on several different mechanisms. One vision is that kiss-and-run fusion occurs when the threshold for full fusion is not reached. The threshold would be determined by the interplay between the regenerative recruitment of SNARE-mediated force and some kind of restraining force that counterbalance vesicle fusion. According to this “restraining force hypothesis” cytoskeleton-dependent restraining barriers exist that counterbalance SNARE-driven fusion mechanisms (113). Studies in neurons have shown that the relative incidence of kiss-and-run fusion is strongly regulated by key factors such as intracellular calcium accumulation, impulse frequency, and a previous history of activity. Calcium is important to switch the kiss-and-run mode of secretion to the full fusion events. High calcium shifts the mechanism from kiss-and-run to complete fusion (120). Furthermore, some lines of evidence suggest that the number of SNARE complexes might also be important in the balance between kiss-and-run and full fusion. In reconstituted systems (121), it has been calculated, for example, that one SNARE complex is sufficient for the bilayer fusion observed in kiss-and-run and three of those complexes are needed to prevent the nascent fusion pore from reclosing (a phenomenon needed in full fusion exocytosis). Also, the stability of the fusion pore can be altered by accessory proteins of SNARE function, including Syt (122), complexin (123), and Gβγ (124).

## Endocytosis–Exocytosis Coupling

Regardless of the mechanism of exocytosis, the incorporation of membrane during exocytosis has to be closely coupled to endocytosis to retrieve the additionally inserted membrane. Several modes of endocytosis have been characterized (125). They include endocytosis after (i) full-collapse fusion, in which collapsed vesicles are retrieved by classical endocytosis involving membrane invagination and vesicle reformation, (ii) kiss-and-run fusion, in which the fusion pore just opens and closes without any significant membrane traffic, and (iii) after compound/multivesicular exocytosis one can also observe bulk endocytosis that retrieves giant vesicles from the membrane. In agreement with the multiple types of exocytosis in MC, a recent electrophysiological study has shown that, in addition to classical mode endocytosis, kiss-and-run as well as compound exocytosis events can be delineated (126).

Following exocytotic fusion the formed SNARE complexes are also reconstituted into the individual SNARE proteins. This is achieved by the NSF ATPase (127) and represents the energy consuming step within the SNARE cycle (98). The necessity of NSF in MC exocytotic event has been demonstrated by the fact that transfection of an inactive NSF mutant into RBL MC blocks degranulation (63).

## *De novo* Secretory Pathways

Besides secreting mediators prestored in cytoplasmic granules, MC release also a whole array of *de novo* synthesized mediators. These include lipid compounds such as leukotrienes and prostaglandins, which are generated from arachidonic acid released from nuclear membrane phospholipids through the action of cytosolic phospholipase A2. These compounds are synthesized in the cytosol and then diffuse across the PM due to their lipid-derived nature and hence do not require lipid transport mechanisms (128).

MC also synthesize and release a large set of different cytokines, growth factors, and chemokines. An extensive list produced by MC can be found in a review by Galli and coworkers (4). Contrary to the lipid mediators, they are proteins and synthesized at the rough ER and released along the secretory pathway using vesicular carriers (31). As indicated certain cytokines and growth factors such as TNF and VEGF have also been shown to be present in cytoplasmic granules and thus can also be released by anaphylactic degranulation providing an immediate source available within minutes (4, 42). On the contrary *de novo* synthesized cytokines and chemokines require several hours to obtain maximal levels of secretion engaging complex signaling pathways. They involve transcriptional regulation through transcription factors, epigenetic control mechanisms, as well as post-transcriptional regulation through mRNA stabilization and microRNA (miRNA). Signaling pathways also exist at the level of vesicular trafficking regulating the selective sorting to specific small vesicles and tubovesicular organelles. The relative contribution of these control mechanisms remains to be clarified but could largely differ between individual cytokines and chemokines. Some of the important signaling steps leading to their secretion are summarized in the following chapters.

### Transcriptional control by the activation of transcription factors

**Figure 4:**
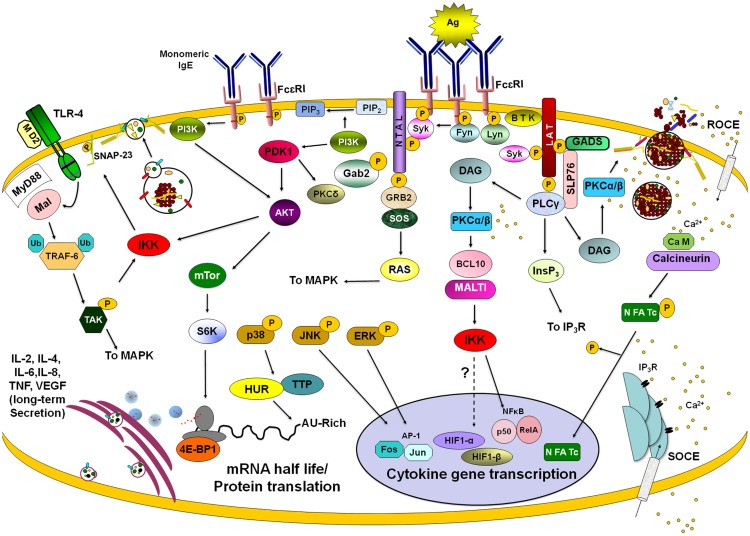
**Late signaling events in *de novo* cytokine/chemokine production**. Stimulation of different membrane receptors lead to the activation of transcription factors, modulators of mRNA turnover, and effectors of ribosome activity that provoke long-lasting secretion of lymphokines in mast cells. After FcεRI triggering with IgE/antigen (Ag) complexes, Lyn and Fyn tyrosine kinases become activated and phosphorylate ITAMs from β and γ chains of the receptor. Further activation of Syk kinase leads to the phosphorylation of a number of adapters (such as LAT and NTAL) and enzymes (such as BTK and PLCγ). Enzymes and adapters form different macromolecular aggregates leading to the activation of different signaling pathways. PLCγ activation is connected with PKCα/β-dependent events through the generation of DAG and IP3. PKCα/β connect FcεRI triggering with IKK activation and IκB degradation, leading to nuclear translocation of NFκB. IP3-sensitive calcium release from intracellular stores lead to the activation of a store-operated Ca^2+^ entry (SOCE). IgE/Ag stimulation initiates also a receptor-operated Ca^2+^ entry (ROCE). Calcium increase allows calcineurin-dependent dephosphorylation and nuclear translocation of NFAT. On the other hand, Fyn-PI3K pathway leads to the activation of IKK and NFκB-dependent transcription but also triggers AKT, which controls mTor kinase and the initiation of protein translation by S6K and 4E-BP1. Activation of MAPK provokes the activation of AP-1 transcription factor but also modifies cytokine mRNA turnover through the modulation of mRNA-binding proteins such as HUR and Tristetraprolin (TTP). Binding of monomeric IgE to FcεRI induces mTor-dependent changes on 4E-BP1 phosphorylation leading to modifications on cap-dependent protein translation. Stimulation of toll-like receptor 4 (TLR4) leads to NFκB-mediated transcription by the MyD88/Mal-dependent pathway, which renders IKK activation after the TRAF-6/TAK1 axis. IKK, either activated by FcεRI or TLR4 receptors, phosphorylates SNAP-23 facilitating secretion. Also, IKK triggering (together with some calcium-dependent signals) modulates HIF1-α-dependent transcription. Once translated, cytokines and angiogenic factors are secreted for variable periods of time utilizing specific vesicle carriers. See text for details.

Figure 4 displays some of the important signaling pathways controlling *de novo* synthesis of cytokines in MC. Some of the details of the early signaling events leading to the activation of Ca^2+^ mobilization and PKC via PLCγ and DAG have already been described above. This PLCγ-DAG-Ca^2+^ signaling then initiates a signaling wave that culminates in the activation of different transcription factors for cytokine/chemokine production. Important transcription factors include nuclear factor of activated T cells (NFAT), nuclear factor-kappa B (NFκ-B), and activator protein-1 (AP-1), but many other transcription factors may also be involved depending on the cytokine/chemokine gene.

Nuclear factor of activated T cells are a family of four transcription factors (NFAT1–4) that in normal conditions are phosphorylated and reside in the cytoplasm (129). MCs have been shown to express NFAT1 and 2 (130). In stimulated cells, NFAT becomes dephosphorylated by calcineurin, a Ca^2+^-calmodulin-dependent serine/threonine phosphatase. This results in a conformational change that now exposes a nuclear localization sequence (NLS), which binds importins, allowing NFAT translocation to the nucleus and initiation of the transcription of a number of pro-inflammatory and regulatory cytokine genes, such as IL-2, IL-4, IL-13, and TNF (130, 131). The rephosphorylation of NFAT then exposes a nuclear export sequence (NES) enabling transport back into the cytosol via the exportin Crm1.

Another essential transcription factor regulating cytokine expression in MC is NFκB (132). This family of proteins includes five members: NFκB1 (p50), NFκB (p52), RelA (p65), RelB, and cRel. Although they can form different homo and heterodimers, p50/cRel is the most common form of NFκB in immune cells (133). In MC, as in other immune cells, the activity of NFκB is controlled by inhibitor of kappa B (IκB) protein that binds to the heterodimer in the cytoplasm maintaining it in an inactive conformation. Activation of NFκB occurs when the IKK phosphorylates IκB on serine residues to target it to proteasomal degradation after polyubiquitination (133). FcεRI-dependent activation of NFκB in MC depends on the activation and lipid raft recruitment of the classical isoform of PKCβ (134) and on the formation of a complex formed by B cell lymphoma 10 (Bcl10) and mucosal-associated lymphoid tissue 1 (Malt1) (135). The PKCβ–Bcl10–Malt1 complex is necessary for NFκB activation and cytokine gene transcription, but not for secretion of pre-formed mediators in MC. Bcl10- and Malt1-deficient MC show normal MAPK (p38, ERK, and JNK), PKC and Akt activation after FcεRI crosslinking but IKK-induced phosphorylation of IκBα and its degradation is completely blocked (135). NFκB is essential for the synthesis of an important number of cytokines in MC including TNF and IL-6 (136).

Besides binding PKC DAG also binds to RAS-guanyl nucleotide-releasing protein (Ras-GRP). Together they activate MAPK and IKK, leading to the activation of the AP-1 (Fos-Jun) and nuclear factor κB (NFκB) transcription factors, respectively (137, 138). AP-1 consists of homo- and hetero-dimers of Jun family proteins, as well as heterodimers of Fos an Jun (139). Fos and Jun proteins are also synthesized and activated by phosphorylation (140). AP-1 acts together with NFAT and NFκB for synergistic activation of cytokine genes (129) for example after costimulation of MC with antigen and IL-33 (141).

Interestingly, NFAT, AP-1, and NFκB are optimally activated in response to different patterns of Ca^2+^ signaling in T cells. Transient high Ca^2+^ spikes lead to sustained activation of JNK and NFκB, but not NFAT, whereas, prolonged low increases in [Ca^2+^]i, were sufficient to activate NFAT (142). Furthermore, as already mentioned above release characteristics depends on the strength of stimulus and this also holds true the activation of NFAT in MC (143).

### Post-transcriptional control of cytokine production

Post-transcriptional regulatory mechanisms play an important role in the regulation of cytokine gene expression (144). They depend in part on specific mRNA sequences present in the 3′ untranslated region (3′ UTR). One major class consists of a conserved AU-rich sequence element (ARE) composed of several repeats of the pentanucleotide AUUUA present in mRNAs encoding growth factors, oncoproteins, or cytokines (145). The ARE has been associated with both an accelerated degradation of mRNA and interference with translation (146). Investigations in mice lacking the TNF ARE sequence in the mouse genome have clearly shown that the TNF ARE was required both for the alleviation and reinforcement of message destabilization and translational silencing in stimulated cells. Moreover, the mutant mRNA was no longer responsive to translational modulation by p38 and JNK kinases, demonstrating that the TNF ARE is a target for these signals (147). One major regulator in this mechanism is TTP, the prototype of a CCCH-zinc finger proteins able to bind to the ARE and to interact with a number of proteins able to regulate mRNA stability and translational control (148). In unstimulated cells, TTP associates with factors that mediate translational repression and mRNA decay, while in stimulated cells with high p38-mitogen-activated protein kinase (p38) and the c-Jun N-terminal/stress-activated protein kinase (JNK/SAPK) activity it associates with proteins that enhance mRNA stability and translation (148).

Cytokine synthesis is also controlled at the level of translation initiation (149). The key signaling pathway controlling the initiation of translation in eukaryotic cells has been shown to be commanded by the mTOR kinase (150) related to lipid kinases and an essential component of two distinct multiprotein complexes named mTOR complex 1 (mTORC1) and mTOR complex 2 (mTORC2) (150). mTORC1, the rapamycin-sensitive complex, consists of mTOR, raptor, and LST8. The mTORC1 signals to inhibitory 4E-binding protein-1 (4EBP-1) and 40S ribosomal protein S6 kinase (S6K), which mediates efficient cap-dependent translation initiation (151). PI3K regulates the mTORC1 pathway via the activation of AKT (151). In MC, IgE-Ag stimulation induces PI3K-dependent activation of mTORC1, S6K1, and 4E-BP1 (52). Inhibition of mTORC1 with rapamycin had no effect on degranulation but inhibited IgE/Antigen-induced IL-6 and IL-8 production and MC survival (52). Interestingly, rapamycin also provoked the destabilization of TNF mRNA in a process that required the TNF ARE in RBL-2H3 cells (152).

Besides cap-dependent translation, mRNAs for some angiogenic mediators, such as the VEGF, can be translated from a structured RNA element termed an internal ribosomal entry site (IRES) able to recruit the 40S ribosomal subunit (153). It has been found that in response to hypoxia and some other stimuli, 4E-BP1 and eIF4G participate in a regulatory pathway that switch protein translation from the normal cap-dependent to the IRES-dependent process (154) allowing selective protein synthesis to cope with an adverse environment. In MC, monomeric IgE induces the release of VEGF from a pre-formed pool but also importantly induces the production of VEGF mRNA by the long-lasting secretion of newly synthesized protein (155). IgE addition to BMMCs leads to dephosphorylation of 4E-BP1, indicating that low-level stimulation of FcεRI is able to induce IRES-dependent translation. The translational switch triggered by IgE was dependent on Fyn kinase activation and correlated with an increase in VEGF mRNA accumulation and VEGF secretion (156).

### microRNAs and cytokine production in MC

microRNAs are a large class of endogenous single-stranded small non-coding RNA molecules that control gene expression by binding to the 3′ untranslated region (3′ UTR) of mRNAs thereby reducing protein synthesis through repression of translation or induction of mRNA degradation. miRNAs control maturation, proliferation, migration, and activation of immune cells (157). MCs express a number of different miRNAs implicated in production of pro-inflammatory mediators. For example, miR-221, originally described to regulate the cell cycle of MC, was shown to favor MC adhesion and migration toward SCF or antigen. This improved IgE/Ag-mediated degranulation and IL-6 and TNF production (158). The miR-146a was found to negatively regulate NFκB signaling, blunting the elevated cytokine production after bacterial infections (159) and contributing to endotoxin tolerance. In MC, miR-146a expression in response to different stimuli is dependent on NFκB p50 (160). MiR-155 expression enhanced FcεRI degranulation and release of TNF, IL-6, and IL-13 related to the activity of the PI3K/Akt pathway (161). Recently, a systematic analysis of miRNA expression during the differentiation of BMMCs lead to the identification of 11 miRNAs that regulate the expression of specific transcription factors and 13 miRNAs that target transcripts of mMCP4 and mMCP6, regulating the synthesis of pre-formed inflammatory mediators (162).

### Epigenetic control of cytokine production in MC

Epigenetic modifications have been shown to influence the synthesis of transcription factors and to modify the sensitivity of promoters including in MC (163). For example, *in vitro* MC differentiation of MC was associated with decreased CD34 expression and increased HIF1A expression compared with bone marrow precursors. These changes were paralleled with changes in the methylation status of the promoters of those genes, suggesting that DNA methylation-dependent epigenetic regulation mediates the gene expression changes involved in maintaining the phenotype of mature MCs together with the differentiation of the HMC-1 cell line (164, 165). In another study, two constitutively DNAse I hypersensitivity sites (HSs) were described within the first intron of the IL-13 gene present in MC regulating the accessibility of the IL-13 locus for high level transcription (166). In the murine MC line derived from fetal liver CFTL-15, a cis-acting element in the second intron of the murine IL-4 gene has a dual function in regulating transcription in MC as well as chromatin accessibility of the IL-4 gene locus through the influence on the methylation state of the gene. MC-restricted transcription factors GATA-1/2 and PU.1 associate with the intron element and regulate its activity (167, 168).

Epigentic modifications are involved not only in the increase on gene transcription but also in the long-term inhibition of cytokine synthesis. In the THP-1 promonocytic cell line, during endotoxin tolerance, it was found that transcription of TNF gene in normal cells was preceded by dissociation of heterochromatin-binding protein 1α, demethylation of nucleosomal histone H3 lysine 9 increased phosphorylation of the adjacent serine 10, and recruitment of NFκB RelA/p65 to the TNF gene promoter. This was no more observed in tolerant cells and RelB, a repressor of transcription, remained bound to the promoter during silencing (169). Since MCs become tolerant after prolonged exposure to LPS, it is possible to speculate that the mentioned mechanisms of TNF gene silencing could also apply.

### Molecular trafficking events in cytokine/chemokine secretion

Contrary to the considerable advances made in the understanding of the regulation of fusion during anaphylactic degranulation, little is still known about the vesicular trafficking involved in MC cytokine/chemokine secretion. One problem is that endogenous cytokines and chemokines are not easily traceable for imaging studies due to the fact that they are secreted over a period of several hours. Thus, for example in macrophages one can observe protein accumulating in the Golgi, while vesicular structures are more difficult to delineate (170). Yet, Stow and coworkers have succeeded to image TNF secretory vesicles in macrophages, which produce considerable amounts of TNF that can be further enhanced by costimulating cells with IFNγ (171). Furthermore, in the case of TNF, addition of a TNF converting enzyme (TACE) inhibitor allows to block cleavage of transmembrane TNF at the PM and to detect its accumulation in the cell surface or in endocytosed vesicular structures (172). In some cases, the authors have also used overexpressed fluorescently tagged cytokines (TNF or IL-6) (173). Interestingly, while continuously produced from the viral promoter, their protein expression further increases after stimulation, which may in part be due to the fact that stimulation leads to enhancement of posttranslational control and vesicular trafficking mechanisms.

Accumulated data suggest that chemokine/cytokine secretion does not follow a unique trafficking pathway but rather could involve multiple pathways and organelles depending also whether they get delivered locally, such as for example at the T cell synapse (174) or the phagocytic cup in macrophages (31) or whether the release is more multidirectional on the cell surface. However, all studies agree that cytokine trafficking differs from the secretion of cytoplasmic SGs containing prestored mediators such as for example shown in NK cells (175).

Most of the vesicular trafficking events have been studied in macrophages. Here, after the initial transport through the Golgi stacks, TNF and IL-6 were found to bud off in highly dynamic tubular–vesicular structures (173). Interestingly, while TNF was generally found together with IL-6, the majority of IL-6 was found in independent carriers. All of them were finally delivered to the ERC, but they seem to reside in distinct non-overlapping areas. From the ERC, the individual cytokines are secreted independently with TNF largely fusing locally at the phagocytic cup, while IL-6 is secreted independently and not targeted to the phagocytic cup. This suggests that the ERC acts as a sorting hub for local secretion of cytokines. Interestingly, examination of another cytokine, IL-10, shows that only about half is delivered to the ERC, while the other half takes another transport route where it is directly delivered from the TGN to the PM via vesicular carriers containing the lipid binding protein ApoE (176). PMD has also been evoked as another mechanism for cytokine secretion. This concerns mostly cytokines prestored in cytoplasmic granules of eosinophils (177).

Concerning the SNARE fusion proteins necessary for constitutive secretion it was found that stimulation of macrophages generally increases their expression, which could be a means to enhance the cytokine trafficking (171, 178). Similarly, some other regulatory proteins such as Rab and Munc18 isoforms are upregulated in LPS-stimulated macrophages (178). The fusion of vesicular carriers arriving from the TGN to the ERC seems to imply a SNARE complex containing the t-SNAREs Syntaxin 6 and Vti1b as well as the ERC-localized v-SNARE VAMP-3. The PM delivery implies a complex composed of SNAP-23 and Syntaxin 4 as well as VAMP-3 (31). Initial analysis of signaling events during such transport indicate that transport is not simply a default mechanism but to the contrary highly regulated. In particular, LPS allows recruitment of PI3Kδ to the TGN, where it allows recruitment of the GTPase dynamin involved in the fission of budding tubulovesicular structures (179). The phosphocholine cytidylyltransferase involved in lipid biogenesis seems also to get activated during this process (31).

Only a few studies have addressed the vesicular trafficking mechanisms in MC. Generally, they point to the fact that, with the exception of cytokines stored in cytoplasmic granules released by anaphylactic degranulation, they are released in a distinct manner that could resemble the mechanisms worked out in macrophages. Indeed, so far none of the SNARE accessory molecules involved in anaphylactic degranulation has shown an implication in cytokine trafficking. Neither the absence of Munc18-2 (70) nor of Syt II (100) has impacted on the secretion of cytokines such as TNF, IL-6, IL-4, or the chemokine CCL2 into the extracellular medium. The latter implies that calcium-mediated regulation may not play a major role in cytokine trafficking although other Syts expressed in MC (7) have not been tested. Yet, secretion of cytokines/chemokines in MC clearly implies SNARE fusion proteins. In human MC, it was shown that introduction of antibodies directed against PM localized t-SNAREs SNAP-23, Syntaxin 3, and Syntaxin 6 could block secretion in permeabilized human MC of all chemokines tested (CCL2, CCL3, CCL4, and CXCL8) albeit it was not always significant in the case of Syntaxin 6 (71). Anti-Syntaxin 4 and anti-VAMP-8 were able to selectively inhibit CXCL8, which could be explained by the fact that CXCL8 is stored in significant amounts in cytoplasmic granules of human MC and hence uses SNARE proteins involved in anaphylactic degranulation of human MC (28). No effects were seen neither for anti-Syntaxin 2, which localizes to the cytosol or the lysosomal marker VAMP-7 (71). In murine MC, functional data with SNARE proteins are not yet available, although the implication of VAMP-3 is suggested by the strong colocalization of VAMP-3 containing vesicles with TNF that had been protected against cleavage by a TACE inhibitor, while no colocalization with VAMP-8 containing vesicles was found (66, 180). This suggests that TNF may in a similar manner to macrophages take route through the ERC. Alternatively, it may also be delivered via the constitutive secretory pathway. Further studies in MC are however necessary to delineate the various vesicular trafficking events involved.

## Diseases Associated with SG Biogenesis and Vesicular Trafficking and Mast Cell Phenotype

In addition to the fundamental studies examining the secretory mechanisms of vesicular trafficking some important information has also been obtained from the study of inherited diseases in humans affecting SG biogenesis and secretory mechanisms in immune cells. Due to the associated defects of lymphocyte cytotoxic functions many of these diseases are characterized by a pathological condition called hemophagocytic lymphohistiocytosis (HLH) with defects in cytotoxic activity and expansion of polyclonal CD8-positive T cells and IFNγ-activated phagocytic macrophages, which infiltrate multiple organs and tissues including the nervous system causing also neurological manifestations [reviewed in Ref. (181)]. Amongst the familial forms of these diseases one can find familial lymphohistiocytosis 1 (FLH1) the most frequent form (50%) with still unknown genotype, FLH2 with a defect in the cytotoxic granule protein perforin, FLH3 with a defect in the SNARE accessory protein Munc13-4, FLH4 with a defect in the t-SNARE syntaxin 11, FLH5 with a defect in the SNARE accessory protein STXBP2 (Munc18-2) (181). Additional diseases showing signs of HLH include Griscelli Syndrome 2 (GS2) with a defect in Rab27A, Chediak–Higashi syndrome characterized by the presence of giant secretory lysosomes and a defect in the Lysosomal trafficking regulator (*Lyst*) gene (181). Another disease complex with secretory phenotypes is the Hermansky–Pudlak syndrome a heterogenous conditions leading to with different types of mutations in genes (*HPS1*, *AP3B1*, *HPS3*, *HPS4*, *HPS5*, *HPS6*, *DTNBP1*, *BLOC1S3*, and *BlOC1S6*) (182). Except for AP3B1 their protein products are part of the Biogenesis of Lysosome-related Organelles Complexes (BLOC), which regulates the traffic of vesicles in the endosomal system and also participate in endosomal membrane secretory lysosome fusion. Clinically the diseases are characterized by platelet secretory dysfunctions with altered granules and oculocutaneous albinism, pulmonary fibrosis, and granulomatous colitis (182). However, only defects in AP3B1, which is part of the AP-3 adaptor complex involved in the formation of new vesicles at the Golgi complex is also associated with lymphohistiocytosis and immune deficiency.

While the MCs phenotype has not been studied in human diseases some knowledge has been obtained from MCs obtained from mice carrying equivalent mutations. We have already described the effect of Munc13-4, Syntaxin 11, Munc18-2, and Rab27 mutations, which except for Syntaxin 11 all show a secretory phenotype. However, this generally concerns only anaphylactic degranulation, while for example cytokine/chemokine production seems not to be affected for example in Munc18-2 deficient cells (see above). Some earlier studies have also analyzed the effect of LYST mutations in MCs obtained from the beige mouse model. LYST codes for a ubiquitously expressed huge (425 kDa) cytosolic protein belonging to the BEACH (beige and Chediak–Higashi) family of proteins, which are proteins involved in vesicular trafficking and synaptic transmission, although their exact function is still unknown. Interestingly, LYST interaction partners include proteins involved, which are part of the ESCRT complex, suggesting a role of Lyst in the formation of intraluminal vesicles. Alternatively, data from the beige mouse model with a defect in LYST or LYST overexpression data indicate that LYST could be important in vesicle generation by fission (183). Indeed, MCs obtained from beige mouse are characterized largely increased giant granules (about 18× the volume), while granules of pancreatic acinar cells showed only a minor increase (23%) (184), suggesting that this may apply specifically to secretory lysosomes as granules from other hematopoietic cells are also increased (183). Furthermore, granule composition appeared to be normal (185). Functional studies also showed that beige MCs were degranulation competent but seemed to show a higher frequency of granule–granule fusion events (186). Electrophysiological studies showed that membrane capacitance increases were about 10-fold higher in beige MCs in agreement with the large granule size (118).

Concerning Hermansky–Pudlak syndrome (HPS) only one elecrophysiological study has been performed with MCs from the ruby-eye mouse showing a defect in HPS6. HSP6 is part of the BLOC-2 complex composed of HSP3, HSP5, and HSP6. Patients with defects in BLOC-2 show a somewhat milder phenotype with absence of the development of pulmonary fibrosis. It interacts with BLOC-1 (*DTNBP1*, *BLOC1S3*, and *BlOC1S6)* required for cargo-specific sorting from early endosomes to lysosome-related organelles (187). In MCs derived from the ruby-eye mouse model a fusion pore phenotype was observed in electrophysiological studies with a threefold increase in the fraction and duration of transient fusion events (188). This suggests that HPS6 and potentially BLOC-2 may also play a direct role in secretion. The increased number of transient fusion events may also be an explanation for the observed platelet granule storage deficiency.

## Concluding Remarks

The secretion of inflammatory mediators by MC through vesicular carriers is a highly regulated process starting with the biogenesis of SGs. Release is triggered by the activation of cell surface receptors initiating signaling processes culminating in the release from cytoplasmic granules by anaphylactic degranulation. As shown in this review many new signaling effectors have been described regulating the various steps involved in SG biogenesis and secretion. *De novo* production of cytokine requires additional control mechanisms at the level of transcriptional and post-transcriptional control of protein synthesis but as new data show may also be regulated at the level of vesicular trafficking, differing from the ones involved in the control of anaphylactic degranulation.

In addition, evidence has shown that MCs are receptive to a number of outside agents activating signaling pathways able to modulate the capacity of MC for secretion. These include, for example immunosuppressive cytokines like the members of the IL-10 family and the TGF-β superfamily (TGFβ and activins) (189). Certain Fc receptors have been also shown to negatively control signaling responses induced by MC (190, 191). Certain GPCRs coupled to Gs proteins that induce the production of cyclic AMP (such as β2-adrenergic, A2, and PGE_2_ receptors), (192) or opiates acting through δ- or μ opiate receptors can also be potent inhibitors of MC secretion (193).

Together, this shows the necessity for exquisite control of the MC secretory processes in order to avoid the dangerous consequences it may have.

## Conflict of Interest Statement

The authors declare that the research was conducted in the absence of any commercial or financial relationships that could be construed as a potential conflict of interest.
